# Probiotic *Clostridium butyricum* CB-a alleviates intestinal inflammation through targeted modulation of the microbiome metabolome axis

**DOI:** 10.1128/spectrum.01351-26

**Published:** 2026-08-04

**Authors:** Jun Liu, Hui Yue, Jiale Li, Zhenyu Fang, Ting Bi, Chenxi Li, Hongbin Yi, Yanhui Zhao, Yanli Feng, Shumiao Zhao, Yuanliang Hu

**Affiliations:** 1 Hubei Key Laboratory of Edible Wild Plants Conservation & Utilization, College of Life Sciences, Hubei Normal University 98425, Huangshi, China; 2 The Fifth Hospital of Huangshi 663817 , Huangshi, China; 3 Xinjiang Tiankang Feed Co., Ltd., Urumqi, China; 4 State Key Laboratory of Agricultural Microbiology and College of Life Science and Technology, Huazhong Agricultural University 124443, Wuhan, China; Karolinska Institutet, Stockholm, Sweden

**Keywords:** *C. butyricum*, ulcerative colitis, gut microbiota, short-chain fatty acids, metabolomics, DSS-induced colitis

## Abstract

This study investigated the capacity of *Clostridium butyricum* CB-a, a novel environmental isolate with unique ecological adaptability, to restore host-microbiome homeostasis in a dextran sodium sulfate (DSS)-induced murine model of intestinal dysbiosis. Integrated 16S rRNA gene sequencing and untargeted LC-MS/MS metabolomics revealed that CB-a (1 × 10⁸ CFU/mL, administered orally) fundamentally restructured the colonic microbial architecture. Specifically, it enriched beneficial, short-chain fatty acid (SCFA)-producing consortia (e.g., *Lactobacillus*, *Bacteroides*, and *Alloprevotella*) while suppressing opportunistic pathobionts (*Escherichia-Shigella*) and mitigating excessive mucin-degrading bacteria (*Akkermansia*). This ecological shift was accompanied by a pronounced metabolic reconfiguration, highlighted by the significant restoration of fecal SCFA pools, predominantly butyrate (*P* < 0.05). Mechanistically, multi-omics correlation potential that the CB-a-driven microbial remodeling alleviates mucosal inflammation through SCFA-linked host-microbe signaling. This pathway explicitly involves the upregulation of G-protein-coupled receptors (GPR41, GPR43, and GPR109A), the inhibition of histone deacetylases (HDAC1/2), and the subsequent reinforcement of epithelial tight junction proteins (ZO-1, Occludin). Furthermore, CB-a significantly attenuated systemic pro-inflammatory cytokine expression while restoring superoxide dismutase (SOD) antioxidant capacity. These findings provide mechanistic insights into how this specific environmental isolate modulates the intestinal microenvironment, offering a robust theoretical basis for deploying *C. butyricum* in functional interventions targeting microbiota-associated inflammatory disruptions.

**IMPORTANCE:**

Severe gut inflammation, such as inflammatory bowel disease, is often driven by a breakdown in our natural gut bacteria. Although probiotics are popular treatments, how they actually repair the gut remains largely unknown. Our study highlights the remarkable healing ability of *Clostridium butyricum* CB-a, a natural bacterium isolated from the environment. We discovered that this microbe acts as an ecological engineer for the digestive system. It actively rescues the damaged gut by promoting the growth of beneficial bacteria and suppressing harmful ones. This positive shift triggers the release of natural, healing molecules that calm the immune system and rebuild the protective gut lining. By uncovering the exact steps this bacterium takes to restore digestive harmony, our work provides a powerful blueprint for designing highly targeted, natural probiotic therapies to combat severe intestinal diseases.

## INTRODUCTION

The mammalian gastrointestinal tract harbors a highly dynamic and intricate microbial ecosystem that is fundamental to host immune regulation and metabolic homeostasis (1). Environmental perturbations, such as profound dietary shifts or chemical stressors, can precipitate severe ecological dysbiosis, which is etiologically linked to the pathogenesis of inflammatory bowel disease (IBD) (2, 3). Rather than viewing IBD solely as a clinical pathology, it represents a fundamental collapse of host-microbiome mutualism. This microecological collapse is typically characterized by a sharp decline in microbial diversity, the depletion of obligate anaerobic short-chain fatty acid (SCFA)-producing consortia, the overgrowth of opportunistic pathobionts, and the subsequent failure of the intestinal epithelial barrier (4, 5). Crucially, this mutualism is governed by the microbiome-metabolome axis, a highly specific, bidirectional communication network. Within this axis, core microbial consortia metabolize substrates into bioactive signaling molecules, most notably SCFAs such as butyrate. Rather than acting merely as energy sources, these metabolites bridge the inter-kingdom gap by binding directly to host target sites, such as G-protein-coupled receptors (GPCRs), and inhibiting enzymes like histone deacetylases (HDACs), thereby continuously tuning mucosal immunity and reinforcing barrier integrity. Consequently, utilizing functional environmental microorganisms to rationally manipulate and restore dysbiotic gut communities has emerged as a compelling paradigm in applied and environmental microbiology (6).

*Clostridium butyricum*, a spore-forming, obligate anaerobic gram-positive bacillus, occupies a versatile ecological niche. It is ubiquitous in diverse natural environments, particularly soil, while also serving as a commensal colonizer in the gastrointestinal tracts of humans and various animals (7). The evolutionary success of *C. butyricum* lies in its exceptional environmental resilience, conferred by its sporulation capacity, and its robust metabolic plasticity, primarily its ability to ferment complex carbohydrates into butyrate and other organic acids. These traits enable environmental isolates of *C. butyricum* to survive harsh extra-host conditions, endure gastrointestinal transit, and effectively colonize the gut to exert profound modulatory effects on the indigenous microbiota (7, 8).

In our previous research, we successfully isolated and characterized a novel environmental strain, *C. butyricum* CB-a, recovered from the feces of free-range chickens residing in mountainous regions. This strain demonstrated superior environmental adaptability, high-density fermentation viability, and notable gastrointestinal tolerance (9). While the basic probiotic applications of *C. butyricum* have been acknowledged, including its approval as a novel food ingredient, the precise multidimensional mechanisms by which a specific environmental isolate mitigates severe host mucosal injury remain incompletely elucidated (10). Specifically, the causal trajectory connecting the introduction of an exogenous environmental strain to the global remodeling of the host microbiome and the subsequent alteration of the metabolic landscape requires comprehensive investigation (11).

Therefore, we hypothesize that the introduction of the environmental isolate *C. butyricum* CB-a can mitigate severe mucosal disruption by driving ecological succession and SCFA-mediated host-microbe signaling. In this study, a dextran sodium sulfate (DSS)-induced murine model was employed not merely to simulate intestinal injury but to represent a severe acute disruption of the intestinal microecology (6). By integrating high-throughput 16S rRNA gene sequencing with untargeted LC-MS/MS metabolomics, this work aims to elucidate the comprehensive host-microbe-metabolite interaction networks orchestrated by *C. butyricum* CB-a, providing a robust mechanistic foundation for its application in microbial ecology engineering and gut health restoration.

## MATERIALS AND METHODS

### Cultivation of *C. butyricum*

The *C. butyricum* strain CB-a is an environmental isolate originally recovered from free-range chicken feces by the School of Life Sciences at Hubei Normal University, with its probiotic characteristics and fermentation profiles detailed in our previous work (9). This strain is preserved in the China Center for Type Culture Collection under the accession number CCTCC M 20242645. The preserved strain was activated in reinforced clostridial medium (RCM) at 37°C for 24 h. Subsequently, 3% (vol/vol) of the activated culture was inoculated into a static fermentation medium and incubated at 34°C for 24 h to obtain the fermented bacterial suspension. The fermentation broth was centrifuged at 2,000 × *g* for 5 min at 4°C to remove the supernatant, and the resulting pellet was washed three times with sterile cold physiological saline (12). The final bacterial suspension was adjusted to a concentration of 1  ×  10⁸ CFU/mL and freshly prepared prior to each experimental use.

### Animal models and treatment

Male KM mice (6–8 weeks old, weighing 18–20 g) were obtained from the Hubei Provincial Laboratory Animal Research Center (Wuhan, China; License No. SCXK(E)2020-0018). The animals were housed under specific pathogen-free (SPF) conditions (temperature: 22°C ± 2°C, relative humidity: 50% ± 10%) with a 12 h light/dark cycle.

### Establishment and disease assessment of the ulcerative colitis mouse model

**Fig 1:**
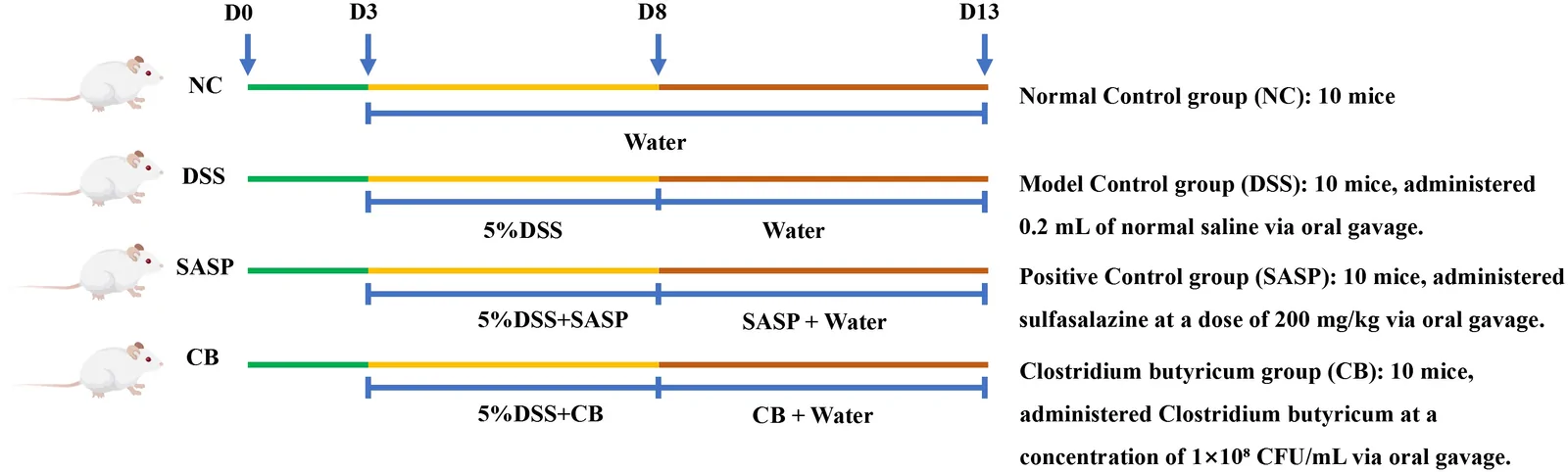
Schematic representation of the experimental design. Mice were administered either 200 mg/kg SASP or *C. butyricum* CB-a from day 4 to day 13 and were treated with or without 5% (wt/vol) DSS during days 4 to 8.

Following a 3-day acclimatization period under laboratory conditions with *ad libitum* access to food and water, 40 male KM mice were randomly divided into four groups (*n* = 10 per group): normal control (NC, administered physiological saline), model control (MC, physiological saline + DSS), positive drug control (SASP, sulfasalazine + DSS), and *C. butyricum* treatment group (CB, *C. butyricum* + DSS). The SASP was administered at a dose of 200 mg/kg, while the CB group received an oral gavage of *C. butyricum* at a standardized daily dose of 2 × 10⁷ CFU/mouse/day (administered via a 0.2 mL volume of a 1 × 10⁸ CFU/mL bacterial suspension). Except for the NC group, all other groups were given 5% DSS in drinking water for 5 days to induce colitis, followed by regular water. Oral administration was performed once daily at a fixed time for 13 consecutive days, with a gavage volume of 0.2 mL per mouse. Mice in the NC and MC groups were gavaged daily with an equal volume of 0.9% physiological saline (Fig. 1) (2, 13).

The disease activity index (DAI) was used to evaluate the progression and severity of colitis induced by DSS, as described in previous studies (13, 14). Parameters assessed included body weight, food and water intake, and stool consistency. At the end of the experiment, mice were fasted for 24 h, followed by euthanasia via cervical dislocation under anesthesia after blood collection via orbital puncture. The spleen, colon, and rectum were carefully excised, surface fat was removed, and organ weights were recorded to calculate the spleen index.

### Serum inflammatory cytokines and antioxidant levels

The serum samples were centrifuged at 3,000 × *g* for 15 min, and the supernatant was collected for further analysis. The concentrations of interleukin-6 (IL-6), interleukin-10 (IL-10), interleukin-1β (IL-1β), and tumor necrosis factor-alpha (TNF-α) in the serum were quantified using enzyme-linked immunosorbent assay (ELISA) kits (Servicebio Biotech Co., Ltd., Wuhan, China) following the manufacturer’s protocols. In addition, the levels of superoxide dismutase (SOD) and malondialdehyde (MDA) in serum were determined using commercial assay kits (Jiancheng Bioengineering Institute, Nanjing, China) in strict accordance with the manufacturer’s instructions (15, 16).

### Hematoxylin and Eosin (H&E) and Alcian Blue–Periodic Acid–Schiff (AB-PAS) staining

Colon tissues were rinsed with phosphate-buffered saline (PBS) and immediately fixed in 4% paraformaldehyde solution for 24 h. Following fixation, samples were dehydrated through a graded ethanol series, cleared with xylene, and embedded in molten paraffin to prepare paraffin blocks. Serial sections of 5 µm thickness were subjected to H&E and AB-PAS staining, respectively. For H&E staining, the rehydrated sections were stained with hematoxylin solution for 3 min, differentiated in 1% acid ethanol for 3–5 s, blued in 0.2% ammonia water for 3–6 s, and counterstained with eosin for 15 s at room temperature. For AB-PAS staining, sections were stained with Alcian Blue solution for 15 min, oxidized in 1% periodic acid for 15 min, and then treated with Schiff’s reagent for 30 min in the dark at room temperature. Histopathological characteristics of the colonic tissue sections were examined using a light microscope (Olympus, Tokyo, Japan) (14).

### Determination of short-chain fatty acids (SCFAs) in feces

Precisely 0.40 g of mouse fecal sample was weighed and mixed with 2 mL of sterile water, vortexed for 30 s, and subsequently placed in an ice bath for 5 min. The mixture was then centrifuged at 12,000 × *g* for 10 min at 4°C. A volume of 1 mL supernatant was collected, supplemented with 10 μL formic acid, and stored at −20°C until analysis. Prior to gas chromatography, 600 μL of the supernatant was filtered through a 0.22 μm organic membrane filter. The quantification of SCFAs was performed using a Shimadzu GC-2010 Plus gas chromatograph (Kyoto, Japan) equipped with an autosampler and flame ionization detector (FID). Separation was achieved using a highly polar DB-WAX 250 fused silica capillary column (30 m × 0.32 mm, 0.25 μm film thickness). The oven temperature program was set as follows: initial temperature at 70°C held for 1 min, increased at 10°C/min to 110°C (held for 1 min), then at 3°C/min to 120°C (held for 1 min), and finally at 3°C/min to 135°C. The injector temperature was maintained at 250°C and the detector at 290°C, with a split ratio of 10:1 and an injection volume of 5 μL. The total run time was 18.66 min. For absolute quantification, standard curves for acetate, propionate, and butyrate were generated using GC-FID peak areas versus known standard concentrations, and SCFA concentrations in fecal samples were calculated from the corresponding regression equations (17).

### Quantitative real-time PCR

Total RNA from colonic tissues was extracted using the BeyoMag Magnetic Bead Animal RNA Extraction Kit (Beyotime Biotechnology, Shanghai, China) according to the manufacturer’s instructions. Following RNA quantification, total RNA (20 ng/μL) was reverse transcribed into complementary DNA (cDNA) using the SweScript RT II First Strand cDNA Synthesis Kit (With gDNA Remover) (Servicebio Biotech Co., Ltd., Wuhan, China). Quantitative real-time polymerase chain reaction (qRT-PCR) was performed using SYBR Green PCR Premix (Servicebio Biotech Co., Ltd., Wuhan, China), and amplification was conducted on a CFX Connect Real-Time PCR Detection System (Bio-Rad, USA). Relative mRNA expression levels were normalized to GAPDH using the 2^−ΔΔCt^ method (3). The sequences of the primers used are listed in Table 1.

**TABLE 1:** Primer sequences used for RT-PCR analyses

Gene (Mus)	Catalog num.	Gene ID	Primer sequence (5′−3′)	Fragment length (bp)
GAPDH (Glyceraldehyde-3-phosphate dehydrogenase)	NM_001411842.1	14433	AGAAGGTGGTGAAGCAGGCATC CGAAGGTGGAAGAGTGGGAGTTG	111
IL-6 (Interleukin 6)	NM_031168.2	16193	TTGTATGAACAACGATGATGCACT TGTTCTTCATGTACTCCAGGTAGC	164
IL-1β (Interleukin 1 beta)	NM_008361.4	16176	GCAACTGTTCCTGAACTCAACT ATCTTTTGGGGTCCGTCAACT	89
TNF-α (Tumor necrosis factor)	NM_001278601.1	21926	GGGCCATAGAACTGATGAGAGGG TCCAGAACTCCAGGCGGT	156
IL-10 (Interleukin 10)	NM_010548.2	16153	GCTCTTACTGACTGGCATGAG CGCAGCTCTAGGAGCATGTG	105
ZO-1 (Zonula Occludens-1)	XM_036152893.1	21872	GCGAACAGAAGGAGCGAGAAGAG GCTTTGCGGGCTGACTGGAG	112
Ocln (Occludin)	NM_001360536.1	18260	TTATACTCCTGCAGACCTGCATCAA GACGGACCCTGACCACTATGAAACA	156
Tff3 (Trefoil factor 3, intestinal)	NM_011575.2	21786	TTGCTGGGTCCTCTGGGATAG TACACTGCTCCGATGTGACAG	117
MUC2 (Mucin 2)	NM_023566.4	17831	ATGCCCACCTCCTCAAAGAC GTAGTTTCCGTTGGAACAGTGAA	101
GPR41 (Free fatty acid receptor 3)	NM_001033316.2	233080	TTCTGAGCGTGGCCTATCCA AGACTACACTGACCAGACCAG	75
GPR43 (Free fatty acid receptor 2)	XM_011250549.4	233079	CTTGATCCTCACGGCCTACAT CCAGGGTCAGATTAAGCAGGAG	137
GPR109A (Hydroxycarboxylic acid receptor 2)	NM_030701.3	80885	CGATTTGGCCGTTCGTTGAA CCTCGCAACCAAAACTGCTC	126
HDAC1 (Histone deacetylase 1)	NM_008228.3	433759	AGTCTGTTACTACTACGACGGG TGAGCAGCAAATTGTGAGTCAT	101
HDAC2 (Histone deacetylase 2)	NM_001428519.1	15182	GGAGGAGGCTACACAATCCG TCTGGAGTGTTCTGGTTTGTCA	173

### LC-MS/MS Analysis

#### Metabolite extraction

Samples were thawed at 4°C, and 50 mg of each sample was accurately weighed and mixed with pre-chilled methanol–water solution (4:1, *v/v*). Homogenization was performed at −10°C using a tissue grinder, followed by a 5 min homogenization step and a subsequent 30 min incubation at −20°C. The mixtures were then centrifuged at 16,000 × *g* for 20 min at 4°C, and appropriate volumes of the supernatants were collected for analysis.

#### Chromatographic separation

Metabolites were maintained in an autosampler at 4°C and analyzed using a SHIMADZU-LC30 ultra-high-performance liquid chromatography system (UHPLC) equipped with an ACQUITY UPLC HSS T3 column (2.1 × 100  mm, 1.8 μm; Waters, Milford, MA, USA). The injection volume was 16 μL, column temperature was set at 40°C, and the flow rate was maintained at 0.3 mL/min. The mobile phases consisted of 0.1% formic acid in water (solvent A) and 0.1% formic acid in acetonitrile (solvent B). The linear gradient elution program was as follows: 0–2 min, 0% B; 2–6 min, B increased from 0% to 48%; 6–10 min, B increased from 48% to 100%; 10–12 min, B held at 100%; 12–12.1 min, B decreased from 100% to 0%; and 12.1–15 min, B held at 0%.

#### Mass spectrometry acquisition

Each sample was analyzed in both positive (+) and negative (–) ion modes using electrospray ionization (ESI). After UPLC separation, metabolites were introduced into a Q Exactive Plus mass spectrometer (Thermo Scientific) equipped with a HESI source. Ionization conditions were as follows: spray voltage, 3.8 kV (+) and 3.2 kV (–); capillary temperature, 320°C; sheath gas flow rate, 3 (arbitrary units); auxiliary gas flow rate, 5 (arbitrary units); probe heater temperature, 350°C; and S-lens RF level, 50. Mass spectrometric data were acquired over a 15-minute run. Full MS scans were collected over an m/z range of 75-–050, with a resolution of 70,000 at m/z 200, AGC target of 3e6, and maximum injection time (IT) of 100 ms. For MS/MS acquisition, data-dependent MS2 scans were triggered for the top 10 most intense precursor ions following each full scan. MS2 acquisition was performed at a resolution of 17,500 at m/z 200, with an AGC target of 1e5, a maximum IT of 50 ms, HCD as the activation type, an isolation window of 2 m/z, and stepped normalized collision energies of 20, 30, and 40.

### 16S rRNA sequencing

Genomic DNA was extracted using the E.Z.N.A. MagBind Soil DNA Kit (Omega Bio-tek, USA) following the manufacturer’s protocol. The V3–V4 region of the 16S rRNA gene was amplified by PCR using specific barcoded primers: 338F (5′-ACTCCTACGGGAGGCAGCA-3′) and 806R (5′-GGACTACHVGGGTWTCTAAT-3′), with genomic DNA as the template. The PCR reaction mixture contained 2 μL of microbial DNA (10 ng/μL), 2 μL each of forward and reverse primers (10 μM), and 25 μL of 2 × Hieff Robust PCR Master Mix (Yeasen, 10105ES03, China). PCR amplification was carried out under the following conditions: initial denaturation at 95°C for 3 min, followed by 30 cycles. Library preparation was performed using the NEBNext Ultra DNA Library Prep Kit for Illumina (New England Biolabs, USA) according to the standard protocol, and sequencing was conducted on the Illumina NovaSeq 6000 PE250 platform (18, 19).

### Statistical analysis

All experiments were performed in triplicate with biological replicates, and results are expressed as means ± standard deviation (SD) unless otherwise specified. Statistical analyses were conducted using SPSS Statistics version 25.0 (IBM Corp., Armonk, NY, USA), with a significance threshold set at *P* < 0.05. All graphical representations and data visualizations were generated using OriginPro 2022 (OriginLab Corporation, Northampton, MA, USA) and R software (version 4.3.1; R Foundation for Statistical Computing, Vienna, Austria).

## RESULTS

### Effects of *C. butyricum* on DSS-induced colonic inflammatory symptoms

**Fig 2:**
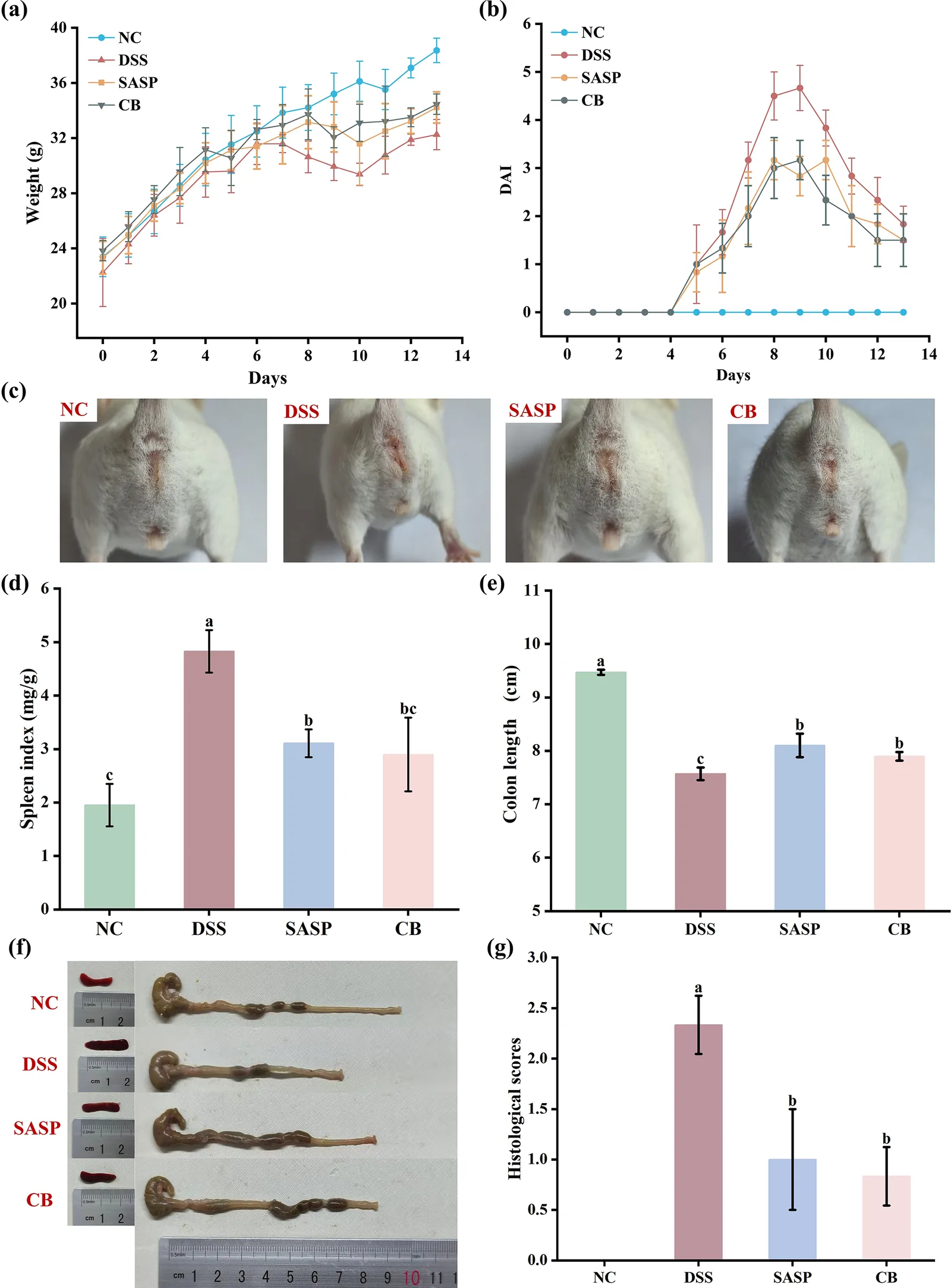
The effects of *C. butyricum* CB-a on DSS-induced ulcerative colitis mice. (**a**) Body weight. (**b**) Disease activity index (DAI). (**c**) Occurrence of anal bleeding on day 13. (**d**) Spleen index. (**e**) Colon length. (**f**) Representative images of the spleen and colon. (**g**) Histological scoring of intestinal pathology sections in mice. NC denotes normal control mice; DSS denotes mice administered 5% DSS alone; SASP denotes mice treated with SASP (200 mg/kg); CB-a denotes mice treated with 1 × 10⁸ cfu/mL CB-a. Data are presented as mean ± SD (*n* = 10). Bars labeled with different superscript letters (**a–c**) denote significant differences (*P* < 0.05), as determined by Duncan’s multiple range test.

As illustrated in Fig. 2a and b, the control group, as expected, exhibited the most pronounced body weight gain and the lowest DAI index. Compared with the other two groups (DSS and SASP), mice fed with CB-a demonstrated superior weight gain and a lower DAI index. Notably, DSS treatment resulted in weight loss by day 7 and a sharp increase in DAI on day 5, indicating that DSS-induced colitis compromised the overall health status of the mice. Figure 2c and f present the morphological characteristics of the anus, colon, and spleen. DSS-treated mice exhibited anal bleeding and deformation, a markedly shortened colon (*P* < 0.05), and splenomegaly accompanied by a significantly elevated spleen index (*P* < 0.05) (Fig. 2d and e). In contrast, both SASP and CB-a treatment significantly ameliorated anal morphology and colon shortening, with the spleen index of the CB-a group showing a 39.96% reduction compared with the DSS group (*P* < 0.05). Collectively, these findings indicate that *C. butyricum* CB-a exerts beneficial effects on the growth and overall health of mice.

### Effects of *C. butyricum* on colonic mucosal integrity and barrier function in DSS-induced colitis

**Fig 3:**
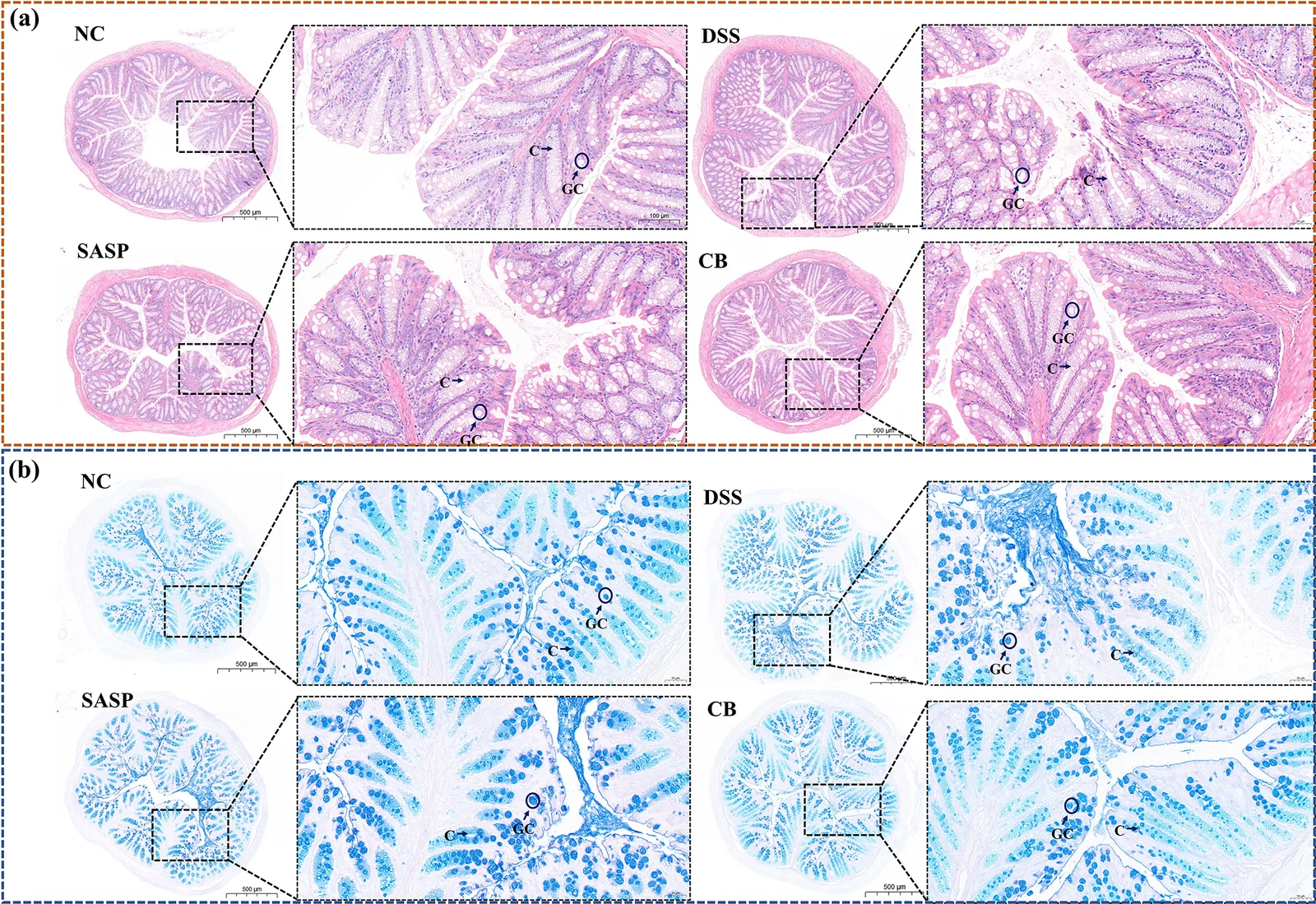
Histopathological alterations of the colon in DSS-induced UC mice following treatment with *C. butyricum* CB-a. (**a**) H&E-stained sections. (**b**) AB-PAS-stained sections. Scale bar = 500 µm. C, crypt; GC, goblet cell; NC, normal control mice.

As illustrated in Fig. 3, colonic tissues from the NC group of mice exhibited typical physiological structural features, with intact organization across all layers of the intestinal wall. The epithelial cells were neatly arranged with good continuity, and no significant inflammatory cell infiltration was observed in either the lamina propria or the submucosa, with no evidence of tissue injury. In contrast, the DSS group demonstrated severe disruption of mucosal integrity, characterized by extensive loss or disorganization of crypt architecture, discontinuity of the epithelial layer accompanied by localized shedding, and massive infiltration of inflammatory cells (including neutrophils and lymphocytes) within the lamina propria and submucosa. Following intervention with SASP or CB-a, colonic tissue injury in both groups was markedly ameliorated compared with the DSS group, as evidenced by partial restoration of mucosal integrity, reduced crypt destruction, and a significant decrease in inflammatory cell infiltration.

AB-PAS staining further revealed that colonic mucosa in the NC group contained abundant goblet cells, which were arranged tightly and regularly along the glands, secreting mucus that effectively covered the epithelial surface and maintained normal barrier function. In contrast, the DSS group displayed a pronounced reduction in goblet cell numbers compared with the NC group, along with disrupted cell arrangement, loss of polarity, and even complete absence of goblet cells in certain regions, indicating substantial impairment of the mucosal barrier due to goblet cell injury in the UC model mice. Treatment with SASP or CB-a significantly increased the number of goblet cells compared with the DSS group, alleviated the extent of disordered arrangement, and markedly reduced histological damage scores (*P* < 0.05) (Fig. 2g). Collectively, these findings suggest that SASP and CB-a promote goblet cell repair and regeneration, thereby restoring colonic mucosal barrier function.

### Effects of *C. butyricum* on serum pro-inflammatory cytokine levels and oxidative status

**Fig 4:**
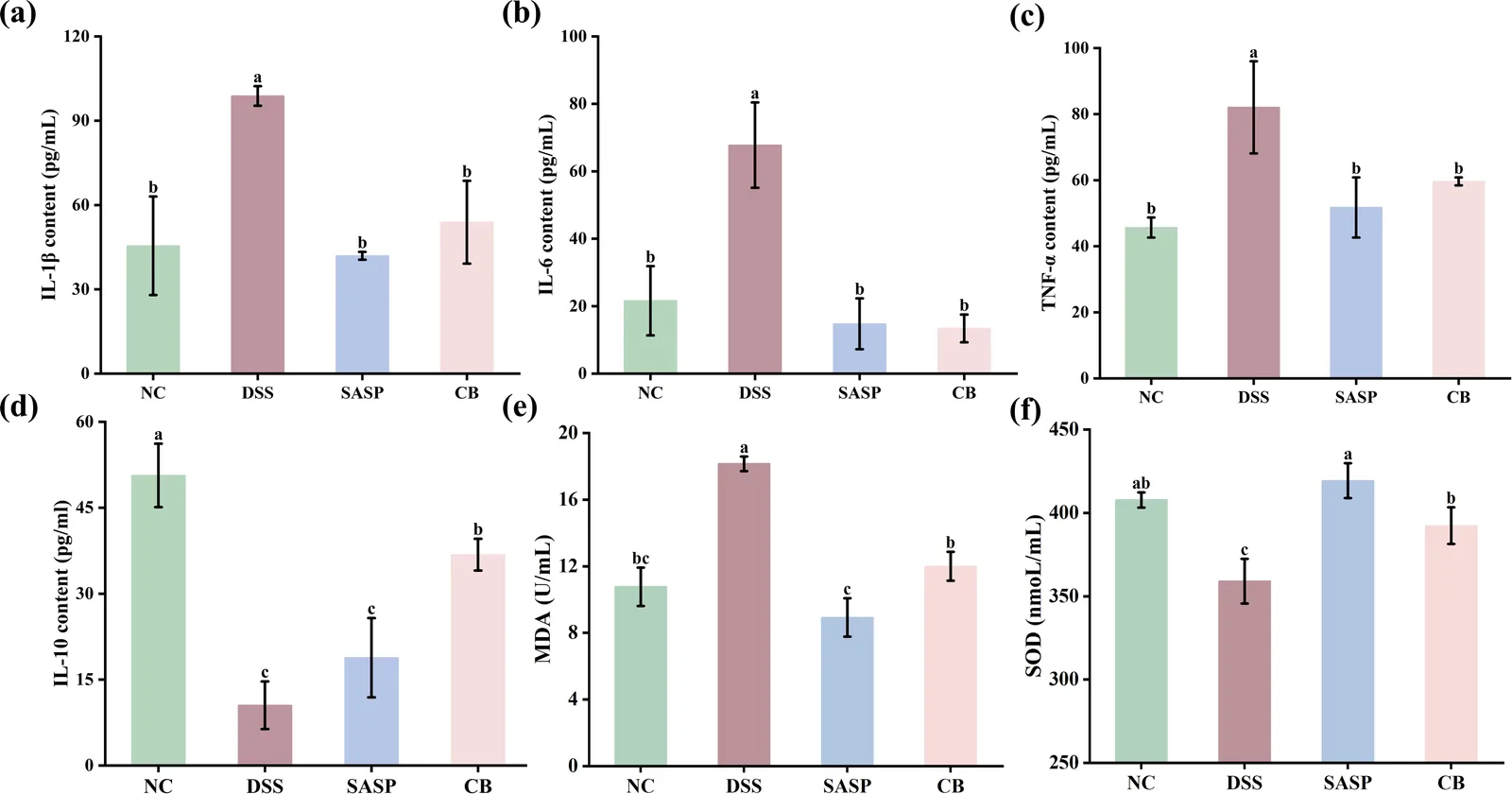
Effects of *C. butyricum* CB-a on serum biochemical parameters and intestinal. (**a**) IL-1β, (**b**) IL-6, (**c**) TNF-α, (**d**) IL-10, (**e**) MDA, and (**f**) SOD activity. Bars labeled with different superscript letters (a–c) denote significant differences (*P* < 0.05), as determined by Duncan’s multiple range test.

Figure 4a through f illustrates the detection results of serum inflammatory cytokines and antioxidant indices in mice. Compared with the NC group, the DSS group exhibited significantly elevated levels of pro-inflammatory cytokines (TNF-α, IL-1β, and IL-6) (*P* < 0.05), with IL-6 in particular increasing by 213.6%. Following SASP and CB-a interventions, the levels of pro-inflammatory cytokines were markedly reduced (*P* < 0.05) (Fig. 4a through c), decreasing by 72.1% and 80.2% relative to the DSS model group, respectively. Regarding anti-inflammatory cytokines, the DSS group displayed a significant reduction in IL-10 compared with the NC group (*P* < 0.05), whereas both SASP and CB-a treatments significantly restored IL-10 levels (*P* < 0.05) (Fig. 4d), showing increases of 79% and 250%, compared to the DSS group, respectively. To further evaluate oxidative stress status, serum superoxide dismutase (SOD) activity and malondialdehyde (MDA) levels were measured. Results revealed that the DSS group showed a significant elevation in MDA compared with the NC group (*P* < 0.05), with an increase of 68.6%. In contrast, both the SASP and CB-a groups exhibited significantly reduced MDA levels (*P* < 0.05) (Fig. 4e). Moreover, SOD activity in the DSS group was significantly lower than in the NC group (*P* < 0.05); however, treatment with SASP and CB-a significantly restored SOD activity (*P* < 0.05) (Fig. 4f).

### Effects of *C. butyricum* on gut microbiota composition

**Fig 5:**
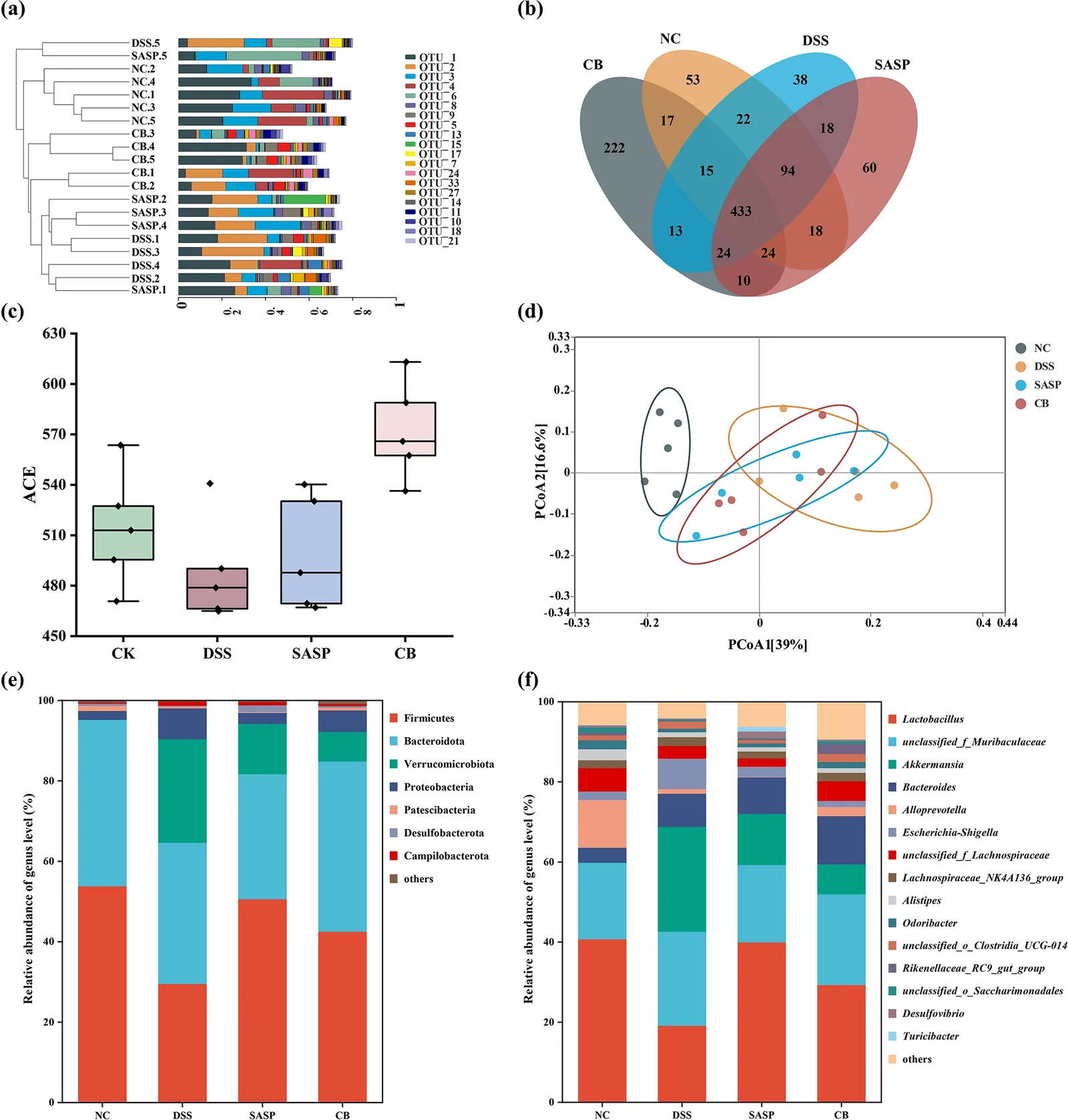
Effects of *C. butyricum* CB-a on the intestinal microbiota of UC mice. (**a**) Phylogenetic tree of OUT levels among groups. (**b**) Venn diagram of the murine intestinal microbiota. (**c**) α-Diversity index based on the ACE metric. (**d**) β-Diversity derived from PCA analysis. (**e**) Relative abundance of the intestinal microbiota at the phylum level. (**f**) Relative abundance of the intestinal microbiota at the genus level. Bars labeled with different superscript letters (a–c) denote significant differences (*P* < 0.05), as determined by Duncan’s multiple range test.

Hierarchical clustering analysis of OTU abundance across the four groups was conducted to evaluate intergroup similarities in microbial community composition (Fig. 5a). Samples within each group exhibited tight clustering patterns, indicating relatively consistent intragroup microbial structures. By contrast, substantial intergroup differences were observed, with the smallest divergence occurring between the NC and CB groups, whereas the NC and DSS groups displayed the greatest dissimilarity (*P* < 0.05). The total number of OTUs in each group was as follows: NC 677, DSS 657, SASP 682, and CB 758 (Fig. 5b). The number of unique OTUs was 38 in the DSS group and 222 in the CB group. α-Diversity analysis based on the ACE index revealed that species richness in the CB group was significantly higher than in the other three groups (*P* < 0.05), while the DSS group exhibited the lowest ACE value (Fig. 5c). Furthermore, β-diversity assessment through principal component analysis (PCA) at the OTU level demonstrated clear segregation of microbial communities among the treatment groups (SASP and CB), the DSS group, and the NC group. Notably, the DSS and NC groups showed the greatest divergence, whereas the CB group exhibited the highest structural similarity to the NC group (Fig. 5d).

The microbial composition of fecal samples at both the phylum and genus levels was determined through OTU abundance analysis. At the phylum level (Fig. 5e), the intestinal microbiota of mice across all groups was primarily composed of *Firmicutes*, *Bacteroidetes*, *Verrucomicrobia*, and *Proteobacteria*. In the NC group, the combined relative abundance of *Firmicutes* and *Bacteroidetes* accounted for 95.2% of the total microbial community. Compared with the NC group, the DSS group exhibited a marked reduction in the abundance of *Firmicutes*, accompanied by significant increases in *Verrucomicrobia* and *Proteobacteria*. Following intervention with SASP or CB-a, the abundance of *Firmicutes* and *Bacteroidetes* rebounded, while *Verrucomicrobia* and *Proteobacteria* declined to levels approaching those of the NC group. At the genus level (Fig. 5f), compared with the NC group, the DSS group showed a significant reduction in the relative abundance of *Lactobacillus*, *Alloprevotella*, Unclassified_f_Lachnospiraceae, *Alistipes*, Rikenellaceae_RC9_gut_group, and Unclassified_O_Saccharimonadales. In the SASP-treated group, the following genera exhibited significant recovery compared with the DSS group: *Lactobacillus*, *Bacteroides*, *Odoribacter*, Rikenellaceae*_*RC9_gut_group, *Desulfovibrio*, and *Turicibacter*. By contrast, CB-a intervention markedly enhanced the abundance of *Lactobacillus*, *Bacteroides*, *Alloprevotella*, Unclassified_f_Lachnospiraceae, *Odoribacter*, Unclassified_O_Clostridia_UCG-014, and Lachnospiraceae_NK4A136_group. On the other hand, DSS treatment led to an increased abundance of *Akkermansia*, *Bacteroides*, *Escherichia-Shigella*, and Unclassified_O_Clostridia_UCG-014. After treatment with SASP or CB-a, the relative abundances of *Akkermansia*, *Escherichia-Shigella*, and Unclassified_O_Clostridia_UCG-014 were reduced to varying degrees, with the CB-a group showing particularly pronounced decreases in *Akkermansia* and *Escherichia-Shigella* (*P* < 0.01).

### Effects of *C. butyricum* on intestinal metabolites

**Fig 6:**
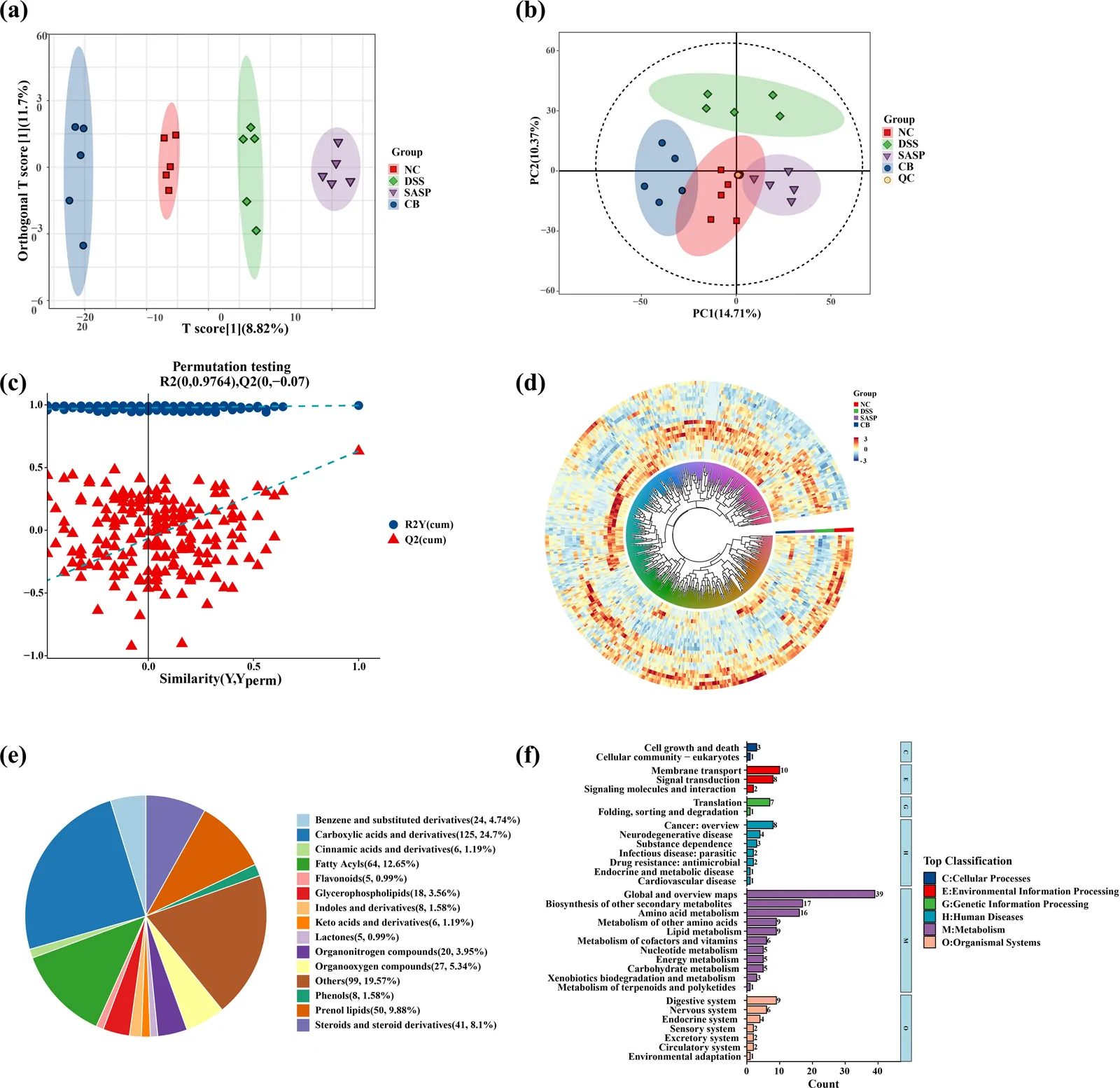
Effects of *Clostridium butyricum* CB-a on intestinal metabolites in UC mice. (**a**) PCA analysis of overall samples under positive and negative ion modes. (**b**) Score plot derived from OPLS-DA analysis. (**c**) Model permutation test. (**d**) Circular heatmap of differential metabolites. (**e**) HMDB Class-based circular diagram of differential metabolite classification. (**f**) KEGG pathways enriched by differential metabolites.

Principal component analysis (PCA) was performed on the metabolites derived from the fecal samples of four groups of mice, and the results are presented in Fig. 6a. Under the mixed positive and negative ion mode, the quality control (QC) samples exhibited tight clustering in the projection space, indicating excellent experimental reproducibility. Distinct clustering patterns were observed among the groups, with the DSS group being completely separated from the NC group, suggesting that DSS treatment markedly perturbed the intestinal metabolic profile of mice. In contrast, the sample points of the SASP and CB-a groups were distributed in regions distinct from the DSS group and were closer to the NC group, implying that both interventions partially reversed the DSS-induced metabolic disturbances. To further enhance the discrimination of intergroup differences, orthogonal partial least squares discriminant analysis (OPLS-DA) was employed. As shown in Fig. 6b, samples from each group displayed clear clustering without overlap, demonstrating pronounced group-specific differences. To evaluate the reliability of the constructed OPLS-DA model and to exclude the risk of overfitting, a permutation test was conducted (Fig. 6c). The results revealed that the *Q*² value of the original model was significantly higher than those obtained from random permutations (with a *Q*² intercept < 0), and the regression line of the permutation test exhibited a negative slope, which conforms to the criteria for model validity. These findings confirm that the model possessed strong predictive capability without signs of overfitting.

Through the screening of metabolites based on variable importance in projection (VIP ≥ 1) and statistical significance (*P* value ≤ 0.05), a total of 634 significantly differentially expressed metabolites (DMs) were identified across the four group comparisons. According to the structural and functional classification system of the HMDB database, the differential metabolites of each comparison group were systematically categorized (Fig. 6d). The results revealed that primary metabolites could be classified into 13 major categories, predominantly including acids and their derivatives (125 species, accounting for 24.70%), prenol lipids (50 species, 9.88%), steroids and steroid derivatives (41 species, 8.10%), and organic oxygen compounds (27 species, 5.34%) (Fig. 6e). Furthermore, pathway enrichment analysis was performed using the KEGG database (Fig. 6f), which demonstrated that these metabolites were significantly enriched in seven functional categories, namely metabolism, genetic information processing, environmental information processing, cellular processes, organismal systems, human diseases, and drug development. Among them, the metabolic category contained the greatest number of annotated metabolites (115 species in total), involving 11 metabolic pathways. Specifically, these included global and overview maps (39 species), biosynthesis of other secondary metabolites (17 species), amino acid metabolism (16 species), metabolism of other amino acids (nine species), and lipid metabolism (nine species).

### Correlation analysis and validation of microorganisms and metabolites

**Fig 7:**
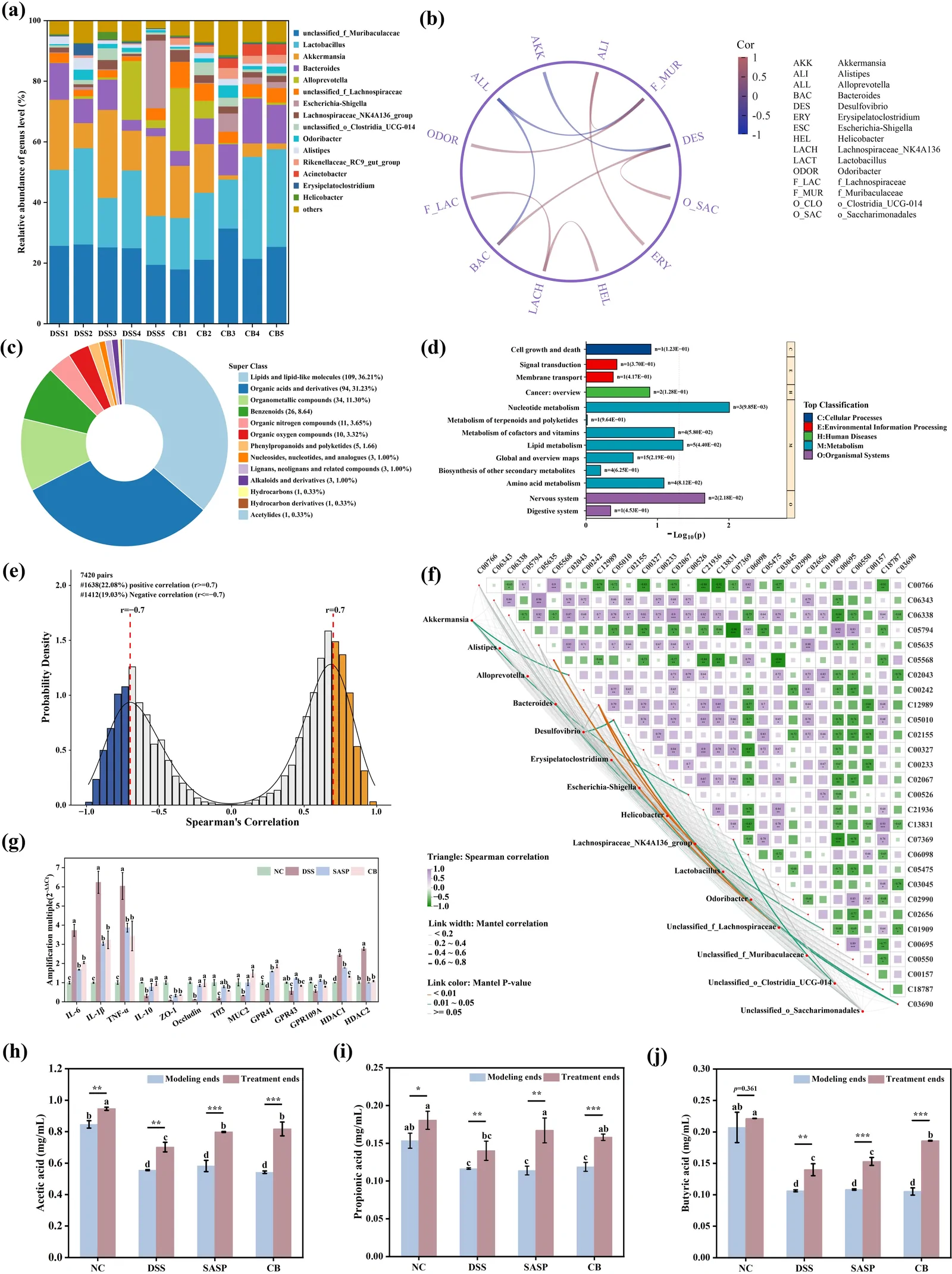
(**a**) Genus-level composition of intestinal microbiota (top 15) in the DSS and CB groups. (**b**) Interaction networks of intestinal microbiota in the DSS and CB groups. (**c**) Functional annotation of metabolites in the DSS and CB groups. (**d**) KEGG metabolic pathways of differential metabolites between the DSS and CB groups. (**e**) Histogram of correlation coefficient (*r*) distributions between microbiota and metabolites in the DSS and CB groups. (**f**) Interactive Mantel test correlation heatmap of microbiota–metabolite associations. (**g**) PCR expression levels of inflammation-related regulatory proteins. (**h**) Acetate levels in mouse feces. (**i**) Propionate levels in mouse feces. (**j**) Butyrate levels in mouse feces. Microbial interactions were considered significant at correlation coefficients ≥ 0.8 with *P* < 0.05. The *x*-axis denotes the r distribution between the two omics data sets, whereas the y-axis represents the density distribution of corresponding *r* values. Generally, *r* ≥ 0.7 or ≤ −0.7 indicates a very strong correlation, as illustrated by the blue (negative correlation) and yellow (positive correlation) regions in the figure. (**a–c**) denote significant differences, and * indicates *P* < 0.05, as determined by Duncan’s multiple range test.

To evaluate the ameliorative effects of *C. butyricum* CB-a on colitis, this study employed the colitis model group as a control to elucidate the underlying mechanisms of CB-a intervention. As shown in Fig. 7a, CB-a treatment markedly suppresses the abundance of *Akkermansia*, *Escherichia-Shigella*, and *Helicobacter*, while simultaneously promoting the proliferation of *Lactobacillus*, *Bacteroides*, and *Alloprevotella*. Among the top 15 most abundant genera, 12 exhibited significant correlations, of which seven were positively associated and four negatively associated. Of particular note, unclassified_f_Muribaculacea*e* and *Desulfovibrio* demonstrated extensive interactions with multiple microbial taxa. For instance, *Bacteroides* displayed a significant negative correlation with *Desulfovibrio* (Fig. 7b). Further metabolomic profiling revealed that lipids and lipid-like molecules and organic acids and derivatives accounted for the largest proportions of differential metabolites, representing 36.21% and 31.23%, respectively (Fig. 7c). KEGG enrichment analysis further indicated that CB-a primarily modulated the global and overview maps and lipid metabolism pathways (Fig. 7d). Histogram analysis delineated the distribution of correlation coefficients and the number of associations exceeding the defined threshold. As shown in Fig. 7e, 22.08% of microbial genera exhibited positive correlations with metabolites, while 19.03% showed negative correlations. Mantel test analysis (Fig. 7f) further demonstrated robust associations between the top 15 genera and differential metabolites annotated with KEGG IDs. For example, *Akkermansia* showed significant correlations with indolyl carboxylic acids and derivatives (C02043) and pyrimidine 2′-deoxyribonucleosides (C00526).

PCR validation further confirmed that CB-a treatment significantly downregulated the expression levels of the pro-inflammatory cytokines IL-6, IL-1β, and TNF-α by 45.06%, 48.11%, and 43.06%, respectively (*P* < 0.05), while markedly enhancing the expression of the anti-inflammatory cytokine IL-10 (increased by 213.87%, *P* < 0.05). Moreover, CB-a intervention significantly upregulated the mRNA expression of intestinal barrier-associated proteins ZO-1, Occludin, Tff3, and MUC2 (*P* < 0.05). The expression of G protein-coupled receptors GPR41 and GPR109, as well as histone deacetylases HDAC1/2, was significantly higher in the CB group compared with other groups (*P* < 0.05) (Fig. 7g). Further analysis of the effects of CB-a on intestinal metabolism in the colitis models is shown in Fig. 7h through j, presenting the changes in fecal short-chain fatty acids (acetate, propionate, and butyrate) across groups. At the end of modeling, compared with the NC group, the levels of all three short-chain fatty acids were reduced in the DSS, SASP, and CB-a groups, although no significant differences were observed (*P* > 0.05). After treatment, acetate levels in the DSS, SASP, and CB-a groups all showed significant recovery compared with baseline, with SASP and CB-a groups exhibiting highly significant increases (*P* < 0.001). Propionate levels in the DSS group did not significantly recover, whereas both SASP and CB-a groups showed significant increases (*P* < 0.05). Butyrate levels were significantly restored in the DSS, SASP, and CB-a groups, with the CB-a group demonstrating an especially marked elevation (*P* < 0.001), reaching 0.186 mg/mL.

## DISCUSSION

Ulcerative colitis (UC) is a globally prevalent intestinal disorder for which no ideal therapeutic strategy has yet been established. Current pharmacological interventions, including aminosalicylic acid derivatives, glucocorticoids, and immunosuppressants, are often associated with various adverse effects (13). Recent evidence suggests that gut microbiota dysbiosis plays a pivotal role in the pathophysiological mechanisms of UC, while the onset and progression of UC further exacerbate microbial imbalance, primarily manifested as abnormal shifts in microbial diversity and abundance (13, 20). Consequently, supplementation with exogenous probiotics to restore intestinal microecological homeostasis has emerged as a promising therapeutic approach for intestinal diseases. Accumulating studies have confirmed that *C. butyricum* exerts inhibitory effects on pathogenic bacteria and demonstrates both preventive and therapeutic efficacy against gastrointestinal infections. Its applications span a broad spectrum of disorders, including acquired intestinal infections, intestinal injury, irritable bowel syndrome, inflammatory bowel disease, metabolic disorders, and colorectal cancer. Owing to its extensive probiotic attributes (20), *C. butyricum* has been recognized as a super probiotic. In the present study, the *C. butyricum* CB-a strain, previously isolated, was employed to evaluate its therapeutic potential in DSS-induced colitis in mice. The experimental findings were consistent with expectations: DSS administration successfully induced severe intestinal injury and inflammatory responses in mice (Fig. 2). Following intervention with *C. butyricum*, colitis symptoms were markedly alleviated, as evidenced by reduced disease activity index (DAI) scores, decreased inflammatory cytokine levels, and restored colonic length. Histopathological analysis further revealed pronounced tissue repair in the colonic mucosa of the treatment group, characterized by well-organized epithelial cell alignment, preserved structural continuity, absence of substantial inflammatory cell infiltration, and uniformly distributed goblet cells at higher densities (Fig. 3).

Building upon the physicochemical analyses, the present study further substantiates the therapeutic efficacy of *C. butyricum* in alleviating intestinal inflammation. Notably, treatment with *C. butyricum* induced pronounced alterations in the gut microbial community structure of mice. In the DSS-induced colitis model, the predominant bacterial genera included *Akkermansia*, *unclassified_f_Muribaculaceae*, *Lactobacillus*, *Bacteroides*, and *Escherichia-Shigella*. Among these, *Akkermansia* and *unclassified_f_Muribaculaceae* dominated the intestinal microbiota, consistent with previous findings suggesting their intimate ecological association with the mucosal layer, characterized by the ability to specifically degrade mucins and mucin-derived glycans (21, 22). Crucially, *Akkermansia* is widely recognized in the field as a beneficial next-generation probiotic under physiological conditions. Its significant enrichment in the present colitis model should not be characterized as an active, pathogenic driver of mucosal injury. Rather, this increased abundance is more accurately interpreted as a passive proliferation, driven by the sudden exposure of mucin-derived carbon sources following the DSS-induced disruption of the intestinal mucus layer (23). In recent years, mucin degradation has been recognized as a central biological process mediating host–microbiota interactions, exerting a “double-edged sword” effect on health and disease. On the one hand, mucin degradation provides microbes with essential carbon, nitrogen, and energy sources, simultaneously producing beneficial metabolites such as SCFAs (24). On the other hand, in a state of severe barrier compromise, excessive degradation by over-colonized taxa can exacerbate the depletion of the mucus barrier and alter the local microenvironment (25, 26), thereby impairing mucosal integrity—findings that are well aligned with the results of this study (Fig. 4). Collectively, these observations suggest an important conclusion: the profound ecological imbalance marked by the overgrowth of mucin-degrading bacteria reflects the severity of endogenous mucosal injury rather than being its primary infectious cause. Distinct from the passive proliferation of mucin-associated taxa, another striking observation was the significant enrichment of opportunistic pathogens such as *Bacteroides* and *Escherichia-Shigella* in the colitis model. As reported by Zafar et al., (27) while *Bacteroides* participates in carbohydrate metabolism, immune modulation, and barrier maintenance, certain species (e.g., *Bacteroides fragilis*) can translocate to extraintestinal sites, causing opportunistic infections such as abscesses and have been implicated in intestinal diseases including colorectal cancer. Similarly, members of the genus *Escherichia-Shigella* are common inhabitants of human and animal intestines. Although *Escherichia coli* is a frequent commensal, its overgrowth can induce diarrhea, whereas *Shigella* is highly infectious and the causative agent of bacillary dysentery (28, 29). The strong diarrheagenic properties of these genera are consistent with the phenotype of bloody stools observed in mice in this study (Fig. 2). Following *C. butyricum* intervention, a substantial remodeling of the intestinal microbiota was observed (Fig. 5), most notably reflected in the dominance of *Lactobacillus*. As a prototypical probiotic genus, *Lactobacillus* is well recognized for its capacity to restore microbial balance, facilitate digestion, enhance immune function, and suppress pathogenic organisms (30). Liu et al. (31) further demonstrated that *C. butyricum* secretes a range of enzymes that degrade carbohydrates into oligosaccharides, which are subsequently metabolized into butyrate, acetate, and other organic acids, thereby creating an acidic microenvironment favorable for lactic acid bacterial proliferation and facilitating probiotic reconstruction. Additionally, microbial remodeling was characterized by the enrichment of SCFA-producing taxa. Notably, four out of the five genera with confirmed probiotic properties exerted their beneficial effects primarily through SCFA biosynthesis; these include Unclassified_f_Lachnospiraceae, *Alloprevotella*, *Lachnospiraceae*_NK4A136_group, and *Odoribacter* (Fig. 7a).

The findings of this study demonstrate that *C. butyricum* ameliorates inflammatory bowel disease through the remodeling of gut microbiota, with its metabolic products, notably butyrate and other SCFAs, playing pivotal roles in intestinal barrier repair and immune modulation. Metabolomic profiling identified a total of 301 differential metabolites, among which lipids and lipid-like molecules as well as organic acids and derivatives were predominant, accounting for 203 metabolites (67.44%). Notably, lipids and organic acids are not metabolically independent entities. SCFAs, as a critical subclass of organic acids, exhibit both commonalities and unique attributes in terms of chemical structure, metabolic origin, and biological functionality (32). KEGG pathway enrichment further underscored that lipid metabolism exerted the most profound influence within microbial metabolic networks. A substantial body of evidence has revealed that SCFAs are central to maintaining intestinal homeostasis and mitigating inflammatory processes. These metabolites are primarily generated by microbial fermentation of dietary fiber and resistant starch, with acetate, propionate, and butyrate constituting the principal SCFAs in the colon (33). According to Zhang et al., SCFAs exert their effects by activating multiple G protein-coupled receptors (GPCRs), including GPR41, GPR43, and GPR109A (also known as PUMA-G). GPR41 and GPR43 are expressed in enteroendocrine cells, mucosal mast cells, and epithelial cells, while GPR43 is predominantly localized in leukocytes, where its activation modulates inflammasome activity, thereby facilitating fiber-mediated intestinal homeostasis (33). Of particular significance, butyrate has been shown to regulate immune cell function via the GPR41 pathway, thereby improving the intestinal microenvironment and promoting mucosal barrier repair (34). Furthermore, GPR109A serves as a highly specific and crucial receptor for butyrate. Its activation on colonic macrophages, dendritic cells, and epithelial cells plays an indispensable role in suppressing intestinal inflammation. By driving the production of anti-inflammatory cytokines, notably IL-10, GPR109A signaling actively fortifies the mucosal barrier against inflammatory insults and facilitates tissue repair (35). Moreover, SCFAs exert inhibitory effects on histone deacetylases (HDACs), thereby attenuating the secretion of pro-inflammatory cytokines and modulating cell growth and differentiation. Their inhibitory potency varies, with butyrate demonstrating the strongest suppression of HDAC1/2 activity (up to 80%), whereas acetate displays only minimal effects (19, 33). Additionally, butyrate regulates macrophage function through HDAC inhibition, leading to reduced tumor necrosis factor (TNF) production and suppression of NF-κB pathway activation (36). Consistent with these mechanisms, our results revealed increased expression of GPR41, GPR43, and GPR109A coupled with decreased HDAC activity in the experimental group, as illustrated in Fig. 7g. Another salient observation was the upregulation of the tight junction protein claudin-1, indicative of improved intestinal inflammation. We posit that this effect is closely associated with SCFAs, particularly butyrate. This inference is corroborated by several studies: for instance, Sumon et al. (2025) reported that butyric glycerides enhance intestinal integrity and barrier function in broilers with necrotic enteritis (32). Huang et al. (2015) demonstrated that sodium butyrate modulates intestinal permeability in pigs by regulating the expression of claudin, occludin, and ZO proteins, thereby reinforcing the mechanical barrier (37). Hu et al. (2024), using a cyclophosphamide-induced intestinal injury model with microbiota transplantation and butyrate supplementation, observed significant upregulation of GPR41, occludin-1, claudin-1, ZO-1, and Muc-2, along with reduced iNOS levels, ultimately facilitating mucosal barrier restoration (34). Collectively, these findings substantiate the hypothesis advanced in the present study.

Metabolomic analysis in the present study revealed that dipeptides, tripeptides, and tetrapeptides exhibited significant differences in the experimental group (CB group), with a pronounced enrichment advantage. The potential origins of these short peptides include the stepwise digestion of dietary proteins in the gastrointestinal tract, microbial metabolic activity, sloughed intestinal epithelial cells, and digestive secretions. Accumulating evidence highlights the multifaceted role of these short peptides in alleviating intestinal inflammation through immune modulation and barrier repair. Rather than acting in isolation, various bioactive peptides have been broadly demonstrated to suppress pro-inflammatory mediators (e.g., TNF-α, IL-6), enhance antioxidant enzyme activities (SOD, GSH-Px), and upregulate essential barrier components like MUC2 and occludin. Furthermore, short peptides can exert direct antimicrobial effects by disrupting bacterial membranes and inducing host endogenous antimicrobial peptide secretion, thereby inhibiting opportunistic pathogens (38–41). Collectively, the significant enrichment of these short peptides following CB-a intervention suggests that they act synergistically with SCFAs to orchestrate host–microbiota interactions, preserve microbial homeostasis, and reinforce barrier functionality. This combined metabolic output provides an additional mechanistic layer explaining how *C. butyricum* CB-a effectively mitigates DSS-induced mucosal injury.

### Conclusions

This study demonstrates that the environmental isolate *C. butyricum* CB-a drives ecological restoration and reestablishes intestinal homeostasis in severe mucosal dysbiosis. By restructuring the colonic microbial community, it enriches bacteria producing short-chain fatty acids and suppresses opportunistic pathobionts. This targeted ecological shift significantly increases butyrate availability. Integrated omics analyses reveal that these metabolites serve as vital signaling molecules coordinating communication between the host and microbiome via GPCR activation and HDAC inhibition to reinforce epithelial integrity. Collectively, these findings provide mechanistic insights into how environmental microorganisms modulate the intestinal microenvironment, highlighting their potential for targeted microbiome engineering. However, limitations remain, including genetic heterogeneity in KM mice and the correlational nature of multi-omics analyses, which require further molecular validation. Future research should explore the colonization dynamics of these isolates across diverse host environments and confirm these mechanistic pathways using targeted *in vivo* knockout models.

## Data Availability

The sequence data generated in this study are publicly available in the NCBI BioProject database under accession number https://www.ncbi.nlm.nih.gov/bioproject/PRJNA1501883. The data supporting the findings of this study are available within the article and its supplemental materials. Additional raw data generated and analyzed during the current study are available from the corresponding author upon reasonable request.
